# Comparison of Conventional and Robotic Fused Filament Fabrication on Silicone Build Plates

**DOI:** 10.3390/ma15186352

**Published:** 2022-09-13

**Authors:** Thomas Herzog, Georg Schnell, Carsten Tille, Hermann Seitz

**Affiliations:** 1 Department of Mechanical, Automotive and Aeronautical Engineering, Munich University of Applied Sciences, 80335 Munich, Germany; 2 Microfluidics, Faculty of Mechanical Engineering and Marine Technology, University of Rostock, 18059 Rostock, Germany; 3 Department Life, Light and Matter, University of Rostock, 18059 Rostock, Germany

**Keywords:** fused filament fabrication, robot, silicone build plate, adhesion strengths, relative shape deviations

## Abstract

The objective of this study is the investigation of the transferability of the material extrusion process from conventional to robotic fabrication on silicone build plates for use in Enhanced Multipoint Moulding with Additive Attachments. Therefore, the study is based on two series of experiments. The first series of tests used a conventional plant extended by a silicone construction platform. In comparison, a six-axis industrial robot was chosen to produce the test specimens in the second series of tests. The comparisons of adhesion strengths and relative shape deviations are used to validate the transferability. The results of the tests show a very good transferability of the process from conventional to robotic production. Whilst angular specimen geometries can be transferred directly, for round specimen geometries, the results show a need for further adaptation to the robot kinematics. The round specimen geometries showed deviations in the surface quality caused by an over-extrusion in the robotic manufacturing. This over-extrusion results from the slicing process in combination with the robot control and may be avoided through further optimisation of the process parameters. Overall, to the best of our knowledge, this study is the first that successfully demonstrates the transfer of Fused Filament Fabrication (FFF) from a conventional system to manufacturing using robots on silicone build plates for the use in Enhanced Multipoint Moulding with Additive Attachments.

## 1. Introduction

In recent years, the number of prototypes required has increased significantly. This increase is caused by a shortening of the model cycles and increasing the individualisation of products. Therefore, the batch size reduces and significantly more moulds are required. As a result of the small series production, these moulds are disposed of after a few production cycles or after just one part is formed. This leads to ecological dilemmas because the almost unused moulds have to be disposed of. Moreover, the costs of the moulds can only be distributed over very few parts. Additionally, there is a long lead time for mould production.

Therefore, Wimmer et al. [1] developed Vacuum-Assisted Multipoint Moulding (VAMM) for Carbon Fibre Reinforced Plastics (CFRP), which is an advancement of the multipoint tooling technology proposed by Cochrane [2]. The main idea is to substitute the production of a rigid mould by setting an adjustable mould. Therefore, the VAMM machine consists of a densely packed and height-adjustable array of pins. By adjusting the height of the individual pins, the mould is set. This results in discrete transitions between the individual pins which are smoothened by a flexible interpolation layer made of silicone.

This setup of the VAMM machine results in certain restrictions in the adjustable geometries. On the one hand, the interpolation layer leads to the intended smoothening of the surface. On the other hand, this results in the impossibility of adjusting transitions with very small radii. Moreover, there are restrictions on the minimal size of the representable mould details as a result of the pin diameter and the thickness of the interpolation layer. Enhanced Vacuum-Assisted Multipoint Moulding with Additive Attachments (EMMA) has been introduced to reduce these restrictions. This process expands the VAMM by additively manufactured attachments representing the parts of the mould which the VAMM could not represent. Due to the exact positioning of the attachments on the interpolation layer, they should be manufactured directly on the silicone-made interpolation layer by Fused Filament Fabrication (FFF), as already outlined by Herzog and Tille [3]. FFF is a material extrusion (MEX) process according to DIN EN ISO/ASTM 52900:2022-03 [4] and DIN EN ISO/ASTM 52903-1:2021-06 [5] and uses a filament as a starting material.

Therefore, FFF has to be performed on a curved silicone build plate. In contrast to the usual glass build plate, the silicone build plate presents an additional challenge for production. Kuo et al. [6] have shown that production on the silicone build plate is possible, but an adhesion promoter is required. Therefore, in Herzog et al. [7], different material and adhesion promoter combinations were investigated for suitability for production on silicone build plates. The study shows that, for the production on a silicone build plate with conventional machines, polylactide (PLA) filament and glue stick as an adhesion promoter are well suited. This research was done on a planar silicone build plate. In contrast, additive manufacturing in EMMA has to take place on a curved silicone build plate. Therefore, Herzog and Tille [3] define the use of an industrial six-axis robot as part of the EMMA. Based on this research, there is an urgent need to investigate the transferability of the former findings to robot-based manufacturing.

The utilisation of industrial six-axis robots for FFF has been shown in different publications. The main aim is the reduction of support structures needed or improved surface quality by production in curved layers in most cases. Wu et al. [8] for example use a flat build plate mounted on a robot arm which allows the division of the component into several parts that can be extruded in the direction of gravity without a support structure. Brooks et al. [9] also mount the build plate on the robot arm, though they use a curved build plate according to the part geometry and then manufacture the part without a support structure. Tam and Mueller [10] also use a curved build plate which is stationary and move the nozzle instead. Li et al. [11] use the robot arm for extruder guidance to enable in-situ application of gel-like materials as a skin substitute. Zhang and Peng [12] and Zhang et al. [13] also use a robot-based manufacturing method and describe the path planning. In combination with rapid cooling of the extrudate, Oxman et al. [14] demonstrate a fully three-dimensional extrusion without a support structure enabled by a robot arm.

Furthermore, Hongyao et al. [15] describe the interaction between the robot and the slicing algorithms to use the bigger build space offered by the robot arm. They also investigate the interaction of multiple robots. Moreover, Yao et al. [16] focus on the manufacturing in one continuous path for the whole part that is enabled by the additional degrees of freedom of the robot arm. Diourté et al. [17] also provide a method for the continuous path planning.

Further investigations, such as Jin et al. [18] and Zhao et al. [19], deal with curved slicing to improve surface quality. In some cases, robot-assisted systems are also used here. In addition, current publications such as Kumar Mishra et al. [20], Kuo et al. [21], Afonso et al. [22], and Devicharan and Garg [23] also focus on the improvement of component quality by varying the manufacturing parameters. At the same time, the improvement of the adhesion on the building platform has also been investigated, e.g., Maidin et al. [24] and Nazan et al. [25].

The former investigations show the possibility of FFF with industrial six-axis robots, although they focus on different improvements. Presently, however, to the authors’ best knowledge, there is no known study of the transferability of the results of conventional FFF production and production using robot arms. Therefore, this paper provides insights into the comparison of both manufacturing methods in the context of the usage in EMMA.

Various evaluation criteria are used to compare the two production methods. First, due to the silicone build plate, adhesion to the build plate is a critical factor that has to be investigated. The adhesion strengths, which represent an adhesive force related to the surface, are used for comparison. This comparison aims to determine whether the different production methods lead to different adhesion strengths. To confirm these findings, a confocal laser scanning microscopy (CLSM) of the printed bottom surface is performed to provide qualitative conclusions about the differences between the manufacturing methods. Particular attention is paid to the topography and the roughness of the adhesion surface as an increasing roughness of the contact surface leads to a higher wettability and therefore influences the adhesion behaviour according to Habenicht [26]. Second, shape deviations are determined using a target/actual approach. Therefore, the geometry of printed specimens is scanned by structured light scanning.

Moreover, the classical portal design suggests a more precise guiding of the nozzle than a serial six-axis industrial robot arm. To determine the influence of this expected loss of guidance accuracy, the deviations in shape between the different manufacturing methods are compared.

## 2. Materials and Methods

### 2.1. Experimental Setup

**Figure 1:**
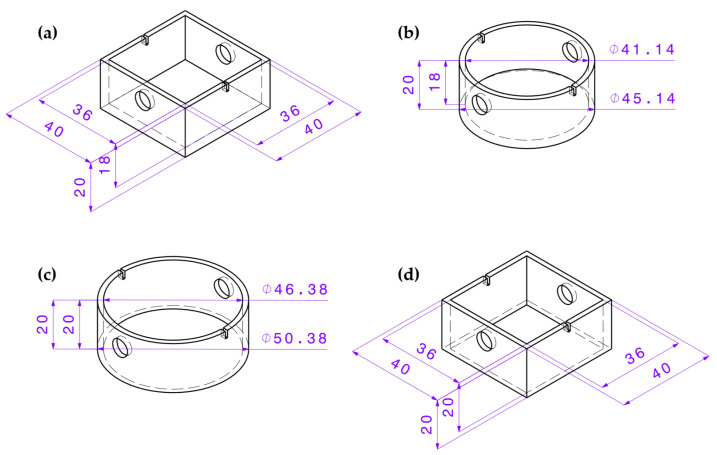
Specimen geometries (**a**) square with base (EG), (**b**) round with base (RG), (**c**) round without base (RO) and (**d**) square without base (EO) with specimen dimensions [7]. Unit: mm.

To determine the specimen geometry influence, the four different specimen geometries as already used in Herzog et al. [7] were tested (Figure 1). There are two square and two round designs. Each of them is designed with and without a base to determine the influence of the difference in the theoretical adhesion area. Within this article, the square geometry with a base is referred to as “EG” (Figure 1a), the square one without a base as “EO” (Figure 1d), the round one with a base as “RG” (Figure 1b) and the round one without a base as “RO” (Figure 1c). The different variants lead to the expectation of different transferability of the results from conventional to robotic production.

Two different test setups were used for the two-test series. For the first test series with the classic commercial FFF machine, the setup consisted of an Ultimaker 3 Extended from Ultimaker B.V., Utrecht, The Netherlands. The production of the specimen was carried out with the Ultimaker print cores of type AA and with a nozzle diameter of 0.4 mm manufactured by Ultimaker B.V., Utrecht, The Netherlands. The standard glass build plate was covered with a layer of the silicone mat. This silicone mat was made of the same material that the interpolation layer of the VAMM machine is made of (Section 2.2). This silicone layer was glued on the glass build plate and fixed with clamps to prevent displacement.

For the second test series, the robot-made specimens were manufactured with the EMMA test rig. This machine consists of the VAMM machine named “full scale prototype” by Wimmer [27]. The VAMM machine consists of 572 height-adjustable pins with a spanner size of 24 mm mounted in a vacuum chamber. These pins form the adjustable mould with a usable size of 400 mm by 600 mm and are traversed by the silicone-made interpolation layer. The interpolation layer consists of five layers of the silicone mat with a thickness of 5 mm. The silicone mats were separated with peel ply to allow relative movement between the layers.

**Figure 2:**
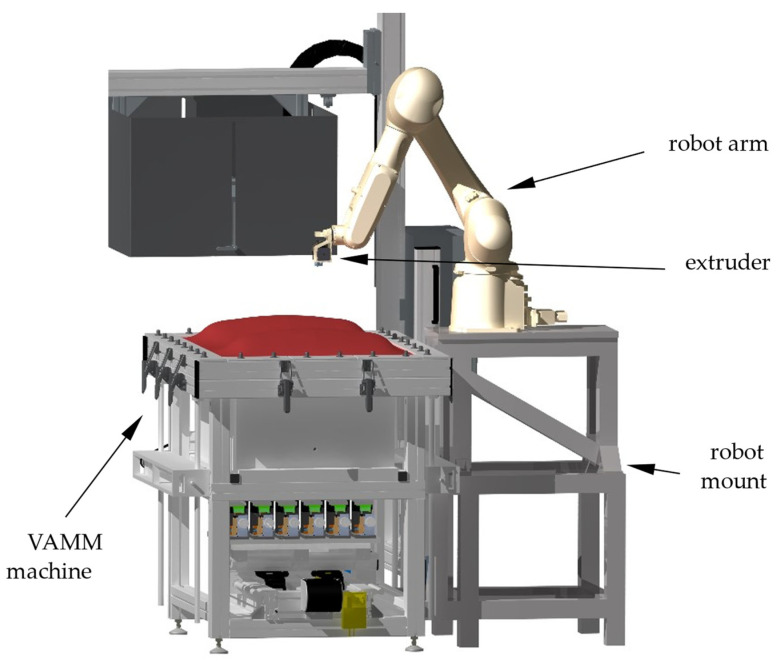
Rendering of the EMMA test rig.

An extruder of the type Titan Aero manufactured by E3D-Online Ltd., Chalgrove, Oxfordshire, United Kingdom, was used to build the specimens. The extruder is mounted on a robot arm of the type RS007LFF60 manufactured by Kawasaki Heavy Industries Ltd., Chūō, Kobe, Japan, which itself is mounted on the VAMM (Figure 2). The robot controller, which is part of the robot arm, operates the extruder’s movement and sends on/off signals to the extruder controller. The extruder controller uses an adapted version of the Repetier software from Hot-World Gmbh & Co. KG, Willich, Germany, running on an Arduino Due from Arduino SA, Chiasso, Switzerland. The extruder controller regulates the nozzle temperature and starts/stops the fixed-speed extrusion depending on the input signal from the robot controller.

For both test series, the same Standard Tessellation Language (STL) files were used as a basis, which were sliced with the standard parameters for PLA in Cura from Ultimaker B.V., Utrecht, the Netherlands. Differing from the standard parameters all layers were printed at the same height for better comparability. Due to the communication limitations between the robot and the extruder controllers, the robot test series was carried out with the same print speed for all areas of the specimen. In contrast, the standard parameter set used in conventional production uses higher print speeds for the less critical areas of the component. The main manufacturing parameters are listed in Table 1. After the slicing, the generated G-Code was directly executed on the conventional machine. The robot cannot execute G-Code; therefore, the Code had to be converted into Kawasaki AS-Language, which was done by a self-programmed converter tool.

**Figure 3:**
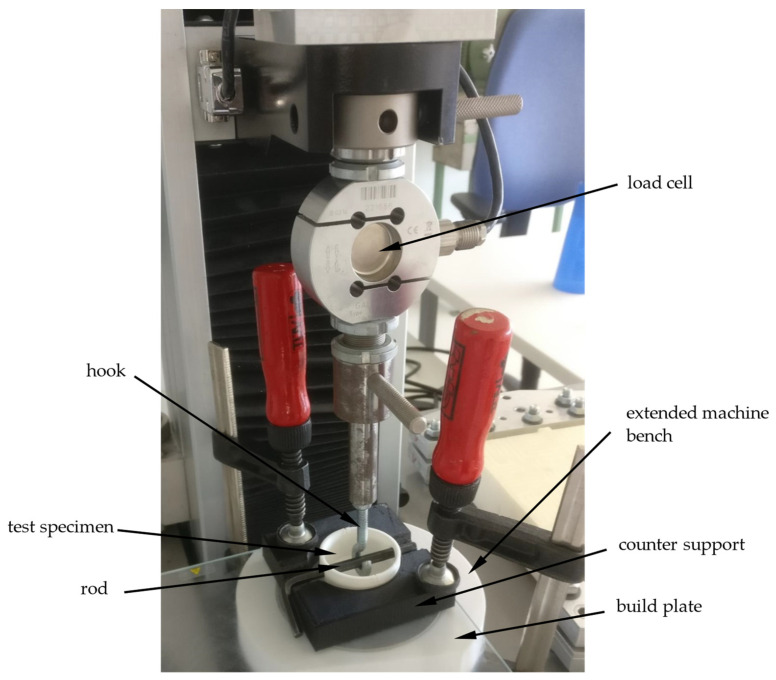
Setup for the tensile test [7].

The adhesive forces of the printed parts on the silicone build plate were determined by a tensile test in the vertical direction. Therefore, a tensile testing machine Quasar 2.5 manufactured by Galdabini SPA, Cardano al Campo, Italy, was used for the specimen produced with the classical machine setup. These tensile tests were carried out with a counter-support enclosing the components (Figure 3) to reduce the deformation of the silicone mat and therefore prevent stress peaks in the adhesion layer. The test specimen was attached to a tension hook with a rod through the openings provided by the specimen (Figure 1). After applying a pre-load of 1 N, the actual tensile test was carried out at a speed of 10 mm/min according to DIN EN 15870 [28].

**Figure 4:**
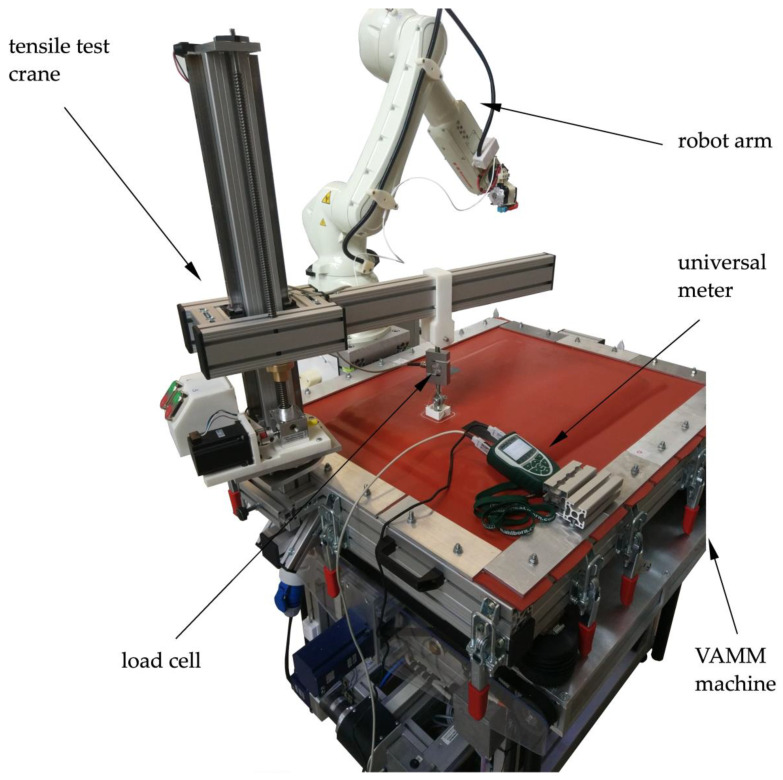
EMMA test rig with the setup for the adhesion test.

As the robot-made specimens are built with the EMMA test rig, the determination of the adhesion forces is not possible by the usage of a conventional tensile test rig. Therefore, a special extension to the EMMA test rig was designed, which allowed the performance of adhesion tests to be undertaken directly on the EMMA test rig. For that purpose, a test crane was mounted on the VAMM machine (Figure 4), which allowed the performance of vertical adhesion strength tests. The test specimens were mounted similarly to the other test series but without a special counter-support. The counter support is built by the vacuum under the interpolation layer. The tensile test was performed with 10 mm/min again, but without the pre-load of 1 N. The measurement was performed by a 1 kN load cell and the associated universal meter ALMEMO 2590 manufactured by Ahlborn Mess- und Regelungstechnik GmbH, Holzkirchen, Germany.

For both test series, the highest measured value is considered as the adhesion force of the specimen. The measured adhesion force is then related to the theoretical adhesion area as adhesion strength according to DIN EN ISO 4624:2006-08 [29].

The shape deviations were determined in the same way for both test series. Therefore, the specimens were at first scanned by structured light scanning. This was performed with a scanner of the type EinScan Pro+ manufactured by Shining 3D Tech Co., Ltd., Hangzhou, China on the associated rotary plate. The shape deviations are determined as a target/actual-comparison with the initial design model in the software GOM Inspect 2019 by GOM GmbH, Braunschweig, Germany. The relative shape deviation is defined in Equation (1) as

(1) $$\Delta_{\mathrm{rel}}=\frac{\mathrm{Integrated}\;\mathrm{absolute}\;\mathrm{distance}}{\mathrm{Area}\;\mathrm{of}\;\mathrm{valid}\;\mathrm{distance}}$$

The integrated absolute distance in Equation (1) represents the absolute volumetric deviation of the manufactured specimen from the target geometry. Using absolute values prevents the mutual cancellation of positive and negative deviations and thus the under-representation of deviations. The area of valid distances is the surface area under comparison. Therefore, the relative shape deviation $\Delta_{rel}$ represents a mean absolute deviation across the whole part.

Furthermore, the surface topography of “EG” and “RG” bottoms was characterized by a Confocal Laser Scanning Microscope (CLSM) of the type LEXT OLS 4000 from Olympus, Hamburg, Germany. Optical magnifications of 10× and 50× were used for visualisation and roughness data collection. Roughness measurements were performed five times per sample at different locations on the surface. Average area surface roughness (Sa) was calculated from achieved 3D data with the software OLS4000 (Olympus, Hamburg, Germany) without a cut-off filter. Both test series were repeated five times. Specimens that differ massively from the target geometry were defined as unsuccessful build specimens. For example, there may be a successfully built first layer, but afterwards, there was a detachment from the build plate, and the next layers are displaced. Regarding the adhesion strength, those failed attempts are added to the analysis with an adhesion strength of 0 N/mm^2^, as all of them were related to unsuccessful bonding on the build plate. Regarding the relative shape deviations, they were not included in the evaluation, since no unsuccessful build samples can be considered to be meaningful. To be clear at this point, this means that an unsuccessful build is included in the evaluation of the adhesion strengths, but not in the relative shape deviations.

### 2.2. Materials

As already mentioned by Herzog et al. [7], the optimised material combination for the production on silicone is Polylactide (PLA) and a glue stick as an adhesion promoter. Therefore, the tests presented in this article were carried out with PLA from Verbatim GmbH, Eschborn, Germany [30]. As an adhesion promoter, the glue stick of the type Stick ecoLogo manufactured by Tesa SE, Norderstedt, Germany [31] was used.

The silicone mats forming the interpolation layer of the VAMM machine and the additional build platelayer for the conventional setup are made of the same material. The type of the material is a VMQ silicone red with a hardness of 40 ± 5 Shore A manufactured by GaFa-Tech Handels GmbH, Schwielowsee, Germany [32].

## 3. Results

**Figure 5:**
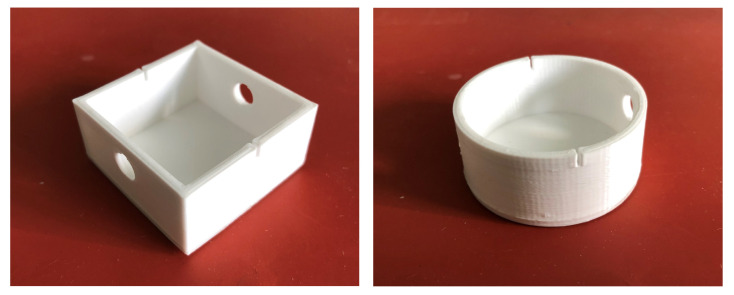
Resulting specimens with conventional production, geometries “EG” and “RG” (taken after the tensile test).

All test specimens were successfully manufactured using conventional FFF. With robot-based FFF, all square specimens were also successfully fabricated, though there were isolated process failures during the production of round specimens. Figure 5 shows two conventionally manufactured test specimens after the tensile test while two robot-produced test specimens are depicted in Figure 10.

**Figure 6:**
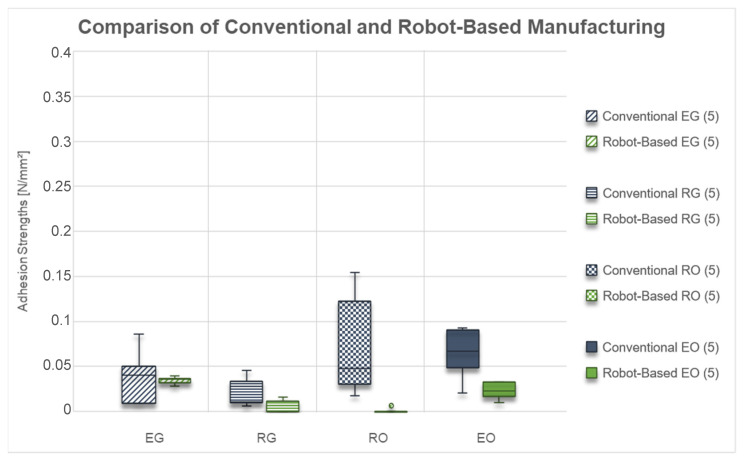
Box Plots of adhesion strengths achieved by conventional and robotic fused filament fabrication on silicone build plates (included number of samples).

The achieved adhesion strengths for conventional and robot-based FFF carried out in this study are shown in Figure 6.

It can be seen that the adhesion strengths for the specimens “EG” are in the same range for both manufacturing methods. While the median values are nearly the same, the adhesion strengths of the conventionally made specimen have a higher scattering. Both manufacturing methods could achieve measurable adhesion strengths for all specimens.

In contrast, the adhesion strengths for the “RG” specimens built with the robot are lower than for conventional manufacturing. Moreover, only three of the five robot-made specimens achieved sufficient adhesion strength. In contrast, the other two could not be built successfully and therefore were counted with an adhesion strength of 0 N/mm^2^.

It is apparent from the “RO” adhesion strengths data that there is an even larger difference between the two manufacturing methods compared to “RG” specimens. The conventionally produced ones could be successfully built all five times. In contrast, only one specimen could be successfully built with the robot and achieve a measurable adhesion strength.

Looking at the “EO” specimens, the differences between the two manufacturing methods are comparable to those achieved with the “EG” geometry. Moreover, for both variants, all specimens could be built successfully and achieved measurable adhesion strengths. However, the adhesion strengths of the “EO” samples for the robot-made ones are lower than for the conventional ones.

Overall, regarding the adhesion strengths, there is a more minor difference when comparing the specimen geometries with or without a base. However, interestingly, there is a large difference between the round specimen and the square ones. The square ones achieve comparable adhesion strengths with both manufacturing methods, whereas the round geometries perform much worse when using the robot. Furthermore, a higher scattering for the conventionally made samples and smaller differences for the robot-made ones can be observed.

**Figure 7:**
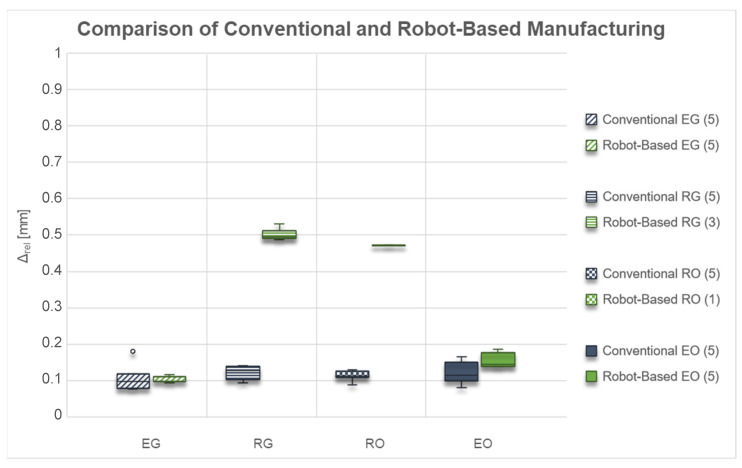
Box Plots of shape deviations by conventional and robotic fused filament fabrication on silicone build plates (included number of samples).

The second evaluation criterion is the shape deviations, which are shown in Figure 7. The shape deviations determined here are the absolute deviations from the nominal geometry related to the specimen surface. This value can be seen as an average deviation of the entire object.

At first glance, similar correlations can be seen with the shape deviations as with the adhesion strengths. While the angular specimens show relatively similar shape deviations for both manufacturing processes, these are significantly higher for the round specimen geometries when manufactured by the robot. It is noticeable that significantly higher shape deviations occur with lower adhesion strengths.

The shape deviations for the test specimens “EG” are almost the same for both production methods, while the scatter is higher for conventional production.

In contrast, the shape deviations for the test specimens “RG” produced with the robot are significantly higher than those from conventional production. At the same time, only three of the five test specimens could be successfully manufactured with the robot and included in the evaluation.

For the test specimen “RO”, an increase in shape deviations can also be observed when switching to robot-production. At the same time, however, the number of successfully manufactured specimens drops again to only one specimen. In the comparison of the adhesion strengths and the shape deviations for conventional production, it is also noticeable that the scatter in the adhesion strengths increases significantly, but in the shape deviations, it is even below that of the other geometries.

For the test specimen geometry “EO”, on the other hand, the shape deviations for the two manufacturing processes are in a comparable range. The slightly lower adhesion strengths of the robot-made specimens lead to slightly higher shape deviations compared to the conventionally produced specimens.

**Figure 8:**
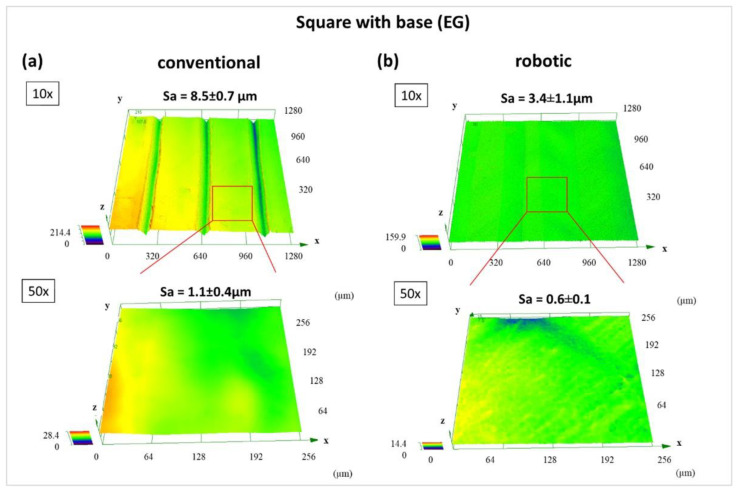
Surface characteristics of “EG”-bottoms determined using CLSM. (**a**) conventional process and (**b**) robotic fused filament fabrication.

**Figure 9:**
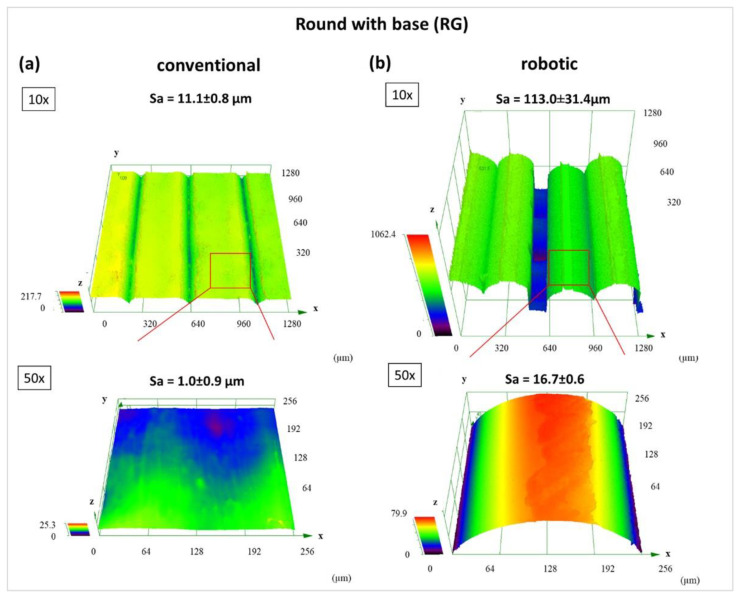
Surface characteristics of “RG”-bottoms determined using CLSM. (**a**) conventional process and (**b**) robotic fused filament fabrication.

CLSM-data are presented in Figure 8 and Figure 9. to compare the difference in surface topography of printed square “EG” and round “RG” samples processed with conventional and robotic FFF. First, no significant differences in surface characteristics of conventional process square and round samples can be overserved (Figure 8a and Figure 9a). Second, the surface topography of robot-made samples appears to be slightly smoother compared to square “EG” samples. However, what stands out in Figure 9 is that the average area surface roughness Sa of the robotically fabricated round “EG” sample is more than ten magnitudes higher than the conventional process round sample. For the specimen geometry “RG”, a significantly coarser strut curve of the bottom surface is noticeable for the robot-made specimens than for the conventionally produced specimens or also for the geometry “EG”. In contrast to the other tests, the individual struts are very clearly visible here, which are also almost round and show only little contact with the building platform.

## 4. Discussion

The aim of this study was the determination of the transferability of FFF from conventional to robot-based FFF. Results show that both the adhesion strengths and the shape deviations show a relatively good agreement between the two manufacturing processes for the square specimens. In contrast, the round specimen geometries show clear differences between the two manufacturing processes. Interestingly, the difference in adhesion strengths of the round samples with both manufacturing methods can be related to the very different bottom topography of the “RG” samples. Furthermore, regarding the decrease in adhesion strength and the increase in relative shape deviations, the construction success also decreases significantly when switching to robot-production. The deterioration of the production result is even more pronounced for the specimen geometry without a bottom (RO) than for the specimen geometry with the bottom (RG). This effect is mainly due to a peculiarity of the robot control in combination with the production parameters and the slicing process.

The first step in the slicing process is converting the designed CAD model into the STL-format. This approximates circular segments of the part by triangular surfaces. The next step is the slicing, in which the body, which is now in the approximated STL format, is divided into individual layers. Based on these layers, the path planning for the nozzle movement takes place. The G-code generated in this process now contains a sequence of short, straight path pieces instead of circular movement commands that would correspond to the original geometry. Compared to three-axis gantry guidance with axes that can be moved independently of each other, the demands on the control system for accurate guidance are significantly higher for serial kinematics such as a six-axis industrial robot. To still meet the accuracy requirements, the robot controller reduces the travel speed to reach the target point. At the same time, there is the option in the controller of superposing between the route to the target point and the subsequent target point. However, the robot controller can only evaluate the point following the current target point and cannot take the further course into account. The short path sections mean that the robot does not reach the target speed because the distance to the last known point, i.e., the next but one, is insufficient. The resulting insufficient movement speed, in conjunction with the specified and fixed extrusion speed, now leads to significant over-extrusion for the round test specimen geometries. However, this problem does not occur with the angular specimen geometries due to the long straight distances between the points.

**Figure 10:**
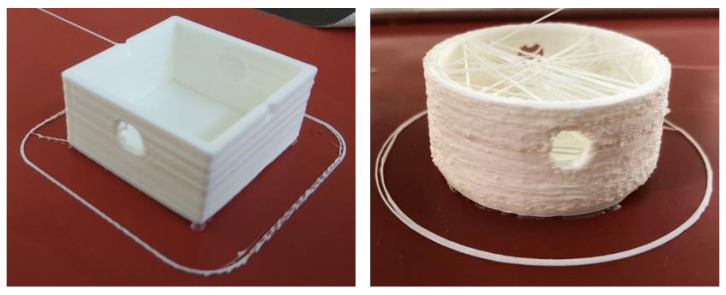
Comparison of the surface quality of the robot-produced test specimens “EG” and “RG”.

**Figure 11:**
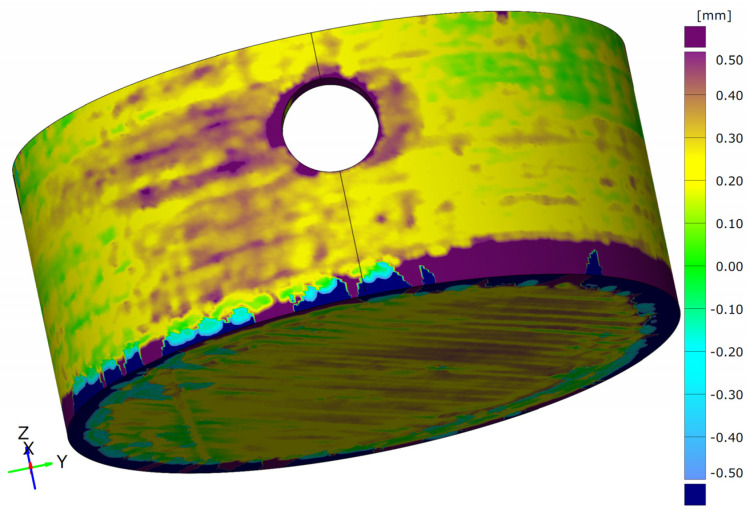
Target/actual surface comparison for the robot-produced test specimen “RG” with the deviations of the rough surface in the lateral component area around the hole as also seen in Figure 10, the soil structure known from the CLSM measurements (Figure 9) and the warping in the edge area.

Figure 10 shows the comparison between a test specimen “EG” and “RG” with the corresponding differences in surface quality by production with the robot. The significantly larger relative shape deviations (Figure 11) are thus already recognisable from the rough, pimply surface of the round test specimen, which is caused by the over-extrusion. At the same time, the over-extrusion also leads to an uneven surface of the respective layer. When passing over these areas in the next layer, the nozzle gets caught in these unevennesses and thus leads to additional stress on the adhesion layer. During the test specimen production, this leads to a weakening of this layer and thus to a loss of adhesion. Depending on the degree of weakening, this can lead to a reduction in the adhesion strength or even to a total loss of adhesion and may result in tearing off the test specimen before it is finished.

Furthermore, the CLSM surface characterisation of the bottom of the robotically made “RG” sample shows a much higher surface roughness than the conventional ones. However, the aforementioned over-extrusion would lead one to expect that the excess amount of material would fill the trenches and create a completely smooth surface. However, the floor surface is created with straight and not circular paths, so that no over-extrusion occurs here. This is due to the significantly lower number of points where the robot reaches its target speed. The same applies to the angular specimen geometries. At the same time, it is also known from the tests that the VAMM has greater deviations in flatness due to the construction of several layers of silicone and the adjustable pins than is the case with the standard construction platform with the silicone mat. Therefore, it can be assumed that, in the area of the building site for the test specimen “RG”, there is also a deepening in the building platform, thus leading to an increased nozzle distance. This in turn leads to the slight under-extrusion in the area of the adhesive surface and thus also to the visible extrusion paths. Then again, on the building site for the angular test specimen “EG”, there might be a slight heightening compared to the build plate of the conventional machine, leading to a more homogeneous adhesion surface. Another small contribution could also result from the robot kinematics, which, due to its principle, tends to be more inaccurate in the z-direction than it is the case with a gantry system. In principle, it would have been conceivable to compensate for the different thicknesses of the silicone surface in the test procedure, but for use in EMMA, taking these unevennesses into account is also the aim of the investigation. These unevennesses occur over the entire VAMM and must therefore be tolerated without manual compensation for a usable EMMA process.

Overall, these issues with the guiding kinematics and the flatness of the VAMM explain the significant difference in the test results between the two production methods for round specimen geometries.

One way to improve the production results for round geometries is to optimise the slicing process so that round geometry parts result in circular travel commands. Another option is to reduce the print speed so that the robot can reach it. At the same time, such a reduction of the print speed also leads to the expectation of a general improvement of the adhesion strengths.

Another disadvantage, especially for the relative shape deviations, of the round test specimens can be seen in Figure 10. Undesired threads are formed inside the specimens. On the one hand, this thread formation results from the dripping of the extruder due to the missing material retraction function. On the other hand, the run on of the extruder also contributes to this thread formation. For the tensile test, these threads had to be removed, so that only residues on the wall found their way into the relative shape deviations. However, these threads do not play a role in the application in the EMMA, as a test specimen with closed surfaces can be assumed for this application, so that thread formation does not occur.

When comparing the two manufacturing processes, however, it can also be seen that the measured values of the robot-manufactured test specimens show less scatter. The most likely explanation for this correlation is the generally lower print speed for the robot-based production. As described above, the first layer was produced at the same travel speed for both manufacturing processes. In the production with the conventional system, a higher travel speed was selected for the other specimen parts. This is impossible with the robot due to the chosen controls, so the entire test specimen was built with a lower travel speed. However, this lower travel speed also leads to lower stresses on the adhesion layer during production. This also results in less pre-damage of the adhesion layer, so that the results are more uniform. This finding is also supported by the slightly smoother surface of the robot-made square compared to the conventionally produced sample.

The last conspicuous feature is the somewhat lower adhesion strengths for the geometry “EO” in robot production, which are within the range of conventional production. In addition to a somewhat higher susceptibility to individual faults in the extrusion due to the smaller floor area, additional unevenness on the build plate can also be considered as a cause. Due to the construction of the “build plate” from the VAMM machine with the adjustable pins and several silicone compounds, a higher tolerance of the build plate flatness inevitably follows.

In general, the findings suggest that production with the robot is certainly comparable with conventional production. The only limitation is the reproduction of the round test specimen geometries, which is, however, due to the peculiarities of the robot control and the path formation. The angular specimen geometries, which circumvent these problems due to their geometry, show the good agreement between the two manufacturing processes. It can therefore be assumed that robot-assisted manufacturing is a suitable approach to combining VAMM and FFF to EMMA. In addition, the robot-assisted manufacturing represents an alternative to producing larger components.

## 5. Conclusions

The purpose of the present study was to investigate the transferability of Fused Filament Fabrication (FFF) from a conventional system to manufacturing using robots for the use in Enhanced Multipoint Moulding with Additive Attachments (EMMA). In EMMA, a curved silicone platform is used for the additively manufactured attachments compared to conventional manufacturing.

Overall, it was shown that the test results can generally be transferred very well from the conventional system to robot-assisted production. However, there are currently limitations on the production of round test specimen geometries with the production parameters adopted from conventional production due to the guiding kinematics of the industrial robot used and the associated control. For the angular test specimen geometries, it was possible to show a very good agreement of the measured values between the manufacturing processes.

Although a very good transferability of the results has already been achieved, there are still some steps to be taken to improve the usability of robot-assisted manufacturing on a large scale. On the one hand, an adaptation to the round geometry parts must be created. With regard to the use of the FFF for EMMA, however, this study was able to demonstrate that the application works with robot-based manufacturing and that a fully functional process should be representable with it.

## Figures and Tables

**Table 1:** General manufacturing parameter of the test specimen for both test series.

Parameter	Value Conventional	Value Robot	Unit
Nozzle diameter	0.4	0.4	mm
Layer thickness	0.2	0.2	mm
Thickness of the first layer	0.2	0.2	mm
Line width	0.4	0.4	mm
Print temperature	200	200	°C
Print temperature of the first layer	200	200	°C
Flow	100	100	%
Flow of the first layer	100	100	%
Print speed of the first layer	20	20	mm/s
Print speed outer walls	50	20	mm/s
Print speed inner walls	55	20	mm/s
Print speed top layer	40	20	mm/s

## Data Availability

The raw data required to reproduce the findings of this published article are available from the corresponding author upon reasonable request.

## References

[1] Wimmer M.S., Lušić M., Maurer C. (2016). Vacuum Assisted Multipoint Moulding—A Reconfigurable Tooling Technology for Producing Spatially Curved Single-item CFRP Panels. Procedia CIRP.

[2] Cochrane J. (1862). Improvement in Presses for Bending Metallic Plates. U.S. Patent.

[3] Herzog T., Tille C. (2021). Review and New Aspects in Combining Multipoint Moulding and Additive Manufacturing. Appl. Sci..

[4] (2022). Additive Fertigung—Grundlagen—Terminologie (ISO/ASTM 52900:2021): Deutsche Fassung EN ISO/ASTM 52900:2021.

[5] (2021). Additive Fertigung—Materialextrusion-basierte additive Fertigung von Kunststoffen—Teil 1: Ausgangsmaterialien (ISO/ASTM 52903-1:2020); Deutsche Fassung EN ISO/ASTM 52903-1:2021.

[6] Kuo C.-C., Chen W.-H., Li J.-F., Zhu Y.-J. (2018). Development of a flexible modeling base for additive manufacturing. Int. J. Adv. Manuf. Technol..

[7] Herzog T., Schnell G., Tille C., Seitz H. (2022). Investigation of suitable material and adhesion promoter combinations for fused filament fabrication on flexible silicone build plates. Rapid Prototyp. J..

[8] Wu C., Dai C., Fang G., Liu Y.-J., Wang C.C. (2017). RoboFDM: A Robotic System for Support-Free Fabrication using FDM. Proceedings of the IEEE International Conference on Robotics and Automation (ICRA).

[9] Brooks B.J., Arif K.M., Dirven S., Potgieter J. (2017). Robot-assisted 3D printing of biopolymer thin shells. Int. J. Adv. Manuf. Technol..

[10] Tam K.-M.M., Mueller C.T. (2017). Additive Manufacturing Along Principal Stress Lines. 3d Print. Addit. Manuf..

[11] Li X., Lian Q., Li D., Xin H., Jia S. (2017). Development of a Robotic Arm Based Hydrogel Additive Manufacturing System for In-Situ Printing. Appl. Sci..

[12] Zhang J.W., Peng A.H. (2012). Process-Parameter Optimization for Fused Deposition Modeling Based on Taguchi Method. Adv. Mater. Res..

[13] Zhang G.Q., Mondesir W., Martinez C., Li X., Fuhlbrigge T.A., Bheda H. Robotic Additive Manufacturing along Curved Surface—A Step towards Free-form Fabrication. Proceedings of the 2015 IEEE Conference on Robotics and Biomimetics.

[14] Oxman N., Laucks J., Kayser M., Tsai E., Firstenberg M., Bártolo H.M., da Silva Bártolo P.J., Alves N.M.F., Mateus A.J., Almeida H.A., Lemos A.C.S., Craveiro F., Ramos C., Reis I., Durão L. (2013). Freeform 3D printing: Towards a sustainable approach to additive manufacturing. Green Design, Materials and Manufacturing Processes, Proceedings of the 2nd International Conference on Sustainable Intelligent Manufacturing, Lisbon, Portugal, 26–29 June 2013.

[15] Hongyao S., Lingnan P., Jun Q. (2019). Research on large-scale additive manufacturing based on multi-robot collaboration technology. Addit. Manuf..

[16] Yao Y., Zhang Y., Aburaia M., Lackner M. (2021). 3D Printing of Objects with Continuous Spatial Paths by a Multi-Axis Robotic FFF Platform. Appl. Sci..

[17] Diourté A., Bugarin F., Bordreuil C., Segonds S. (2021). Continuous three-dimensional path planning (CTPP) for complex thin parts with wire arc additive manufacturing. Addit. Manuf..

[18] Jin Y., Du J., He Y., Fu G. (2017). Modeling and process planning for curved layer fused deposition. Int. J. Adv. Manuf. Technol..

[19] Zhao G., Ma G., Feng J., Xiao W. (2018). Nonplanar slicing and path generation methods for robotic additive manufacturing. Int. J. Adv. Manuf. Technol..

[20] Kumar Mishra P., Ponnusamy S., Reddy Nallamilli M.S. (2021). The influence of process parameters on the impact resistance of 3D printed PLA specimens under water-absorption and heat-treated conditions. Rapid Prototyp. J..

[21] Kuo C.-C., Wu Y.-R., Li M.-H., Wu H.-W. (2019). Minimizing warpage of ABS prototypes built with low-cost fused deposition modeling machine using developed closed-chamber and optimal process parameters. Int. J. Adv. Manuf. Technol..

[22] Afonso J.A., Alves J.L., Caldas G., Gouveia B.P., Santana L., Belinha J. (2021). Influence of 3D printing process parameters on the mechanical properties and mass of PLA parts and predictive models. Rapid Prototyp. J..

[23] Devicharan R., Garg R., Kumar L.J., Pandey P.M., Wimpenny D.I. (2019). Optimization of the Print Quality by Controlling the Process Parameters on 3D Printing Machine. 3D Printing and Additive Manufacturing Technologies.

[24] Maidin S., Wong J., Arif N.M., Mohamed A.S. (2019). Strengthening of Fused Deposition Modeling Printer Bed Adhesion Intensity using ABS Glue. Int. J. Recent Technol. Eng..

[25] Nazan M.A., Ramli F.R., Alkahari M.R., Abdullah M.A., Sudin M.N. (2017). An exploration of polymer adhesion on 3D printer bed. IOP Conf. Ser. Mater. Sci. Eng..

[26] Habenicht G. (2009). Kleben: Grundlagen, Technologien, Anwendungen.

[27] Wimmer M. (2019). Developement, Modelling and Analysis of Vacuum Assisted Multipoint Moulding for Manufacutring of Fibre-Reinforced Plastic Composites. Ph.D. Thesis.

[28] (2009). Klebstoffe—Bestimmung der Zugfestigkeit von Stumpfklebungen: (ISO 6933:1987 modifiziert); Deutsche Fassung EN 15870:2009.

[29] (2016). Beschichtungsstoffe—Abreißversuch zur Bestimmung der Haftfestigkeit: Deutsche Fassung EN ISO 4624:2016.

[30] Verbatim GmbH (2018). PLA 3D Printer Filament.

[31] Tesa SE (2018). Sicherheitsdatenblatt: Tesa Stick + Easy Stick ecoLogo.

[32] GaFa-Tec Handels GmbH (2010). Silikon—Platten.

